# Deep learning based prediction of prognosis in nonmetastatic clear cell renal cell carcinoma

**DOI:** 10.1038/s41598-020-80262-9

**Published:** 2021-01-13

**Authors:** Seok-Soo Byun, Tak Sung Heo, Jeong Myeong Choi, Yeong Seok Jeong, Yu Seop Kim, Won Ki Lee, Chulho Kim

**Affiliations:** 1 Department of Urology, Seoul National University Bundang Hospital, Seongnam, 13620 Korea; 2 Department of Convergence Software, Hallym University, Chuncheon, 24252 Korea; 3 College of Software, Hallym University, Chuncheon, 24252 Korea; 4 Department of Urology, College of Medicine, Hallym University, Chuncheon Sacred Heart Hospital, 153, Kyo-dong, Chuncheon, 24253 Korea; 5 Department of Neurology, College of Medicine, Hallym University, Chuncheon Sacred Heart Hospital, 153, Kyo-dong, Chuncheon, 24253 Korea; 6 Chuncheon Translational Research Center, Hallym University, Chuncheon, 24252 Korea

**Keywords:** Urological cancer, Surgical oncology, Prognosis

## Abstract

Survival analyses for malignancies, including renal cell carcinoma (RCC), have primarily been conducted using the Cox proportional hazards (CPH) model. We compared the random survival forest (RSF) and DeepSurv models with the CPH model to predict recurrence-free survival (RFS) and cancer-specific survival (CSS) in non-metastatic clear cell RCC (nm-cRCC) patients. Our cohort included 2139 nm-cRCC patients who underwent curative-intent surgery at six Korean institutions between 2000 and 2014. The data of two largest hospitals’ patients were assigned into the training and validation dataset, and the data of the remaining hospitals were assigned into the external validation dataset. The performance of the RSF and DeepSurv models was compared with that of CPH using Harrel’s C-index. During the follow-up, recurrence and cancer-specific deaths were recorded in 190 (12.7%) and 108 (7.0%) patients, respectively, in the training-dataset. Harrel’s C-indices for RFS in the test-dataset were 0.794, 0.789, and 0.802 for CPH, RSF, and DeepSurv, respectively. Harrel’s C-indices for CSS in the test-dataset were 0.831, 0.790, and 0.834 for CPH, RSF, and DeepSurv, respectively. In predicting RFS and CSS in nm-cRCC patients, the performance of DeepSurv was superior to that of CPH and RSF. In no distant time, deep learning-based survival predictions may be useful in RCC patients.

## Introduction

Renal cell carcinoma (RCC) is one of the most commonly diagnosed malignant tumors in the urinary tract^1^. The global incidence of RCC increased annually at a rate of approximately 2% during the last two decades^2^. In the United States, new kidney cancer cases and deaths in 2020 were estimated at 73,750 and 14,830, respectively^3^. Most RCC cases frequently diagnosed are surgically resectable tumors with favorable prognoses^4^. Therefore, optimal decision making for postoperative RCC monitoring and provision of better counseling to RCC patients on postoperative prognosis are essential.

Many anatomical, histological, clinical, and molecular markers have been employed for predicting prognosis in RCC patients^2^. However, most of them are difficult to use in clinical settings because they are not easily accessed and analyzable; they lack clear criteria for analysis or use. Therefore, only a few prognostic factors, including the pathological stage, nuclear grade, and histological subtype, are used in clinical practice^2^.

To date, the most prevalent means of predicting prognosis is conducting a survival analysis, which analyzes the time taken for an event such as death or the outcome of interest to occur. The Cox proportional hazard (CPH) model is a standard method of survival analysis that considers the characteristics of an object, such as the effect of the object’s covariates^5^. All of the well-known survival outcomes of diseases, including RCC, have been presented using this CPH model^6–8^.

Because of the widespread emergent use of electronic health record systems, highly sophisticated medical instruments are generating massive amounts of data on patient health daily^9^. As healthcare and medical research gradually transcend the analysis of numerical or structured data, there is an increasing demand for deep learning (DL) analysis of unstructured data such as images and signals^10^. Furthermore, in the handling of unstructured data, DL has the advantage of improving model performance over conventional methods because it can handle large amounts of data speedily and accurately^11,12^. In the field of survival prediction, DL is more accurate than traditional methods, including the CPH model, and has been implemented successfully in the survival analysis of various diseases such as brain tumors, hepatomas, and kidney grafts^13–15^.

To the best of our knowledge, there are no reports on RCC survival prediction using DL using clinical information yet^16^. One articles reported the usefulness of DL algorithm to predict the prognosis of cRCC. However, they used the convolutional neural network to extract the CT/histology imaging markers. Therefore, in our study, we first assessed the prognosis of non-metastatic clear cell RCC (nm-cRCC) using a DeepSurv model in a large multicenter cohort analysis and then compared the results with those obtained using the CPH model.

## Patients and methods

### Patients

This retrospective study was approved by the institutional ethical committee (2019-11-001 at Hallym University Chuncheon Sacred Heart Hospital). Between 2000 and 2014, 2522 nm-RCC patients (pathologically, T1-4N0M0) received curative radical or partial nephrectomy at six institutions in Korea. Among them, 31 underwent lymph node dissection concurrently owing to pre- or intra-operative suspicion of lymph node metastases. However, none had lymph node involvement.

Non-cRCC patients were excluded from the analysis, because their number (n = 383) was insufficient to analyze using DL. Finally, 2139 nm-cRCC patients were included for the analysis.

Table 1 shows the patient variables that were analyzed. Patients were followed up postoperatively every 3 months for the first 6 months, every 6 months for the next 3 years, and annually after that. Recurrence-free survival (RFS) was defined as the time from the surgery date to the date of recurrence, and recurrence was confirmed using imaging modalities. Cancer-specific survival (CSS) was defined as the time from the surgery date to the date of cancer-specific death, and cancer-specific death was confirmed by a physician. Age was determined at the time of surgery. Tumor size was measured using the greatest diameter of the pathological specimen. The pathological stage and nuclear grade were assessed based on the 2009 Tumor-Node-Metastasis classification system and the Fuhrman’s grading system, respectively. The histologic subtype was evaluated based on the 2004 WHO classification. Pathological assessments were performed by urological pathologists in each institution.

**Table 1:** Baseline characteristics between training and test dataset.

	Training dataset (n = 1833)	Test dataset (n = 306)	*p* Value
Age, year	55.4 ± 12.6	56.3 ± 12.7	0.270
Male	1344 (73.3)	203 (66.3)	0.014
BMI, kg/m^2^	24.6 ± 3.2	24.7 ± 3.3	0.576
Diabetes	268 (14.6)	54 (17.6)	0.199
Hypertension	697 (38.0)	110 (35.9)	0.528
**ECOG performance**			
0	1103 (60.2)	255 (83.3)	< 0.001
1–4	730 (39.8)	51 (16.7)	
**Initial symptom**			
Yes	307 (16.7)	85 (27.8)	< 0.001
**Tumor**			
1	1489 (81.2)	233 (76.1)	0.110
2	117 (6.4)	26 (8.5)	
3–4	227 (12.4)	47 (15.4)	
**Fuhrman’s grade**			
1–2	1037 (56.6)	205 (67.0)	0.001
3–4	796 (43.4)	101 (33.0)	
**Tumor size**			
< 40 mm	1039 (56.7)	157 (51.3)	0.137
≥ 40 and < 70 mm	491 (26.8)	98 (32.0)	
> 70 mm	303 (16.5)	51 (16.7)	
Sarcomatoid differentiation	49 (2.7)	7 (2.3)	0.843
Necrosis	94 (5.1)	52 (17.0)	< 0.001
Survived	1745 (95.2)	286 (93.5)	0.253
Survival duration, month	39 (19–66)	47 (11–83)	0.323
Recur free	1675 (91.4)	274 (89.5)	0.348
Recur free duration, month	31 (13–59)	31 (9–71)	0.419

BMI, body mass index; ECOG, Eastern Cooperative Oncology Group.

### Statistical analyses

The data of two largest hospitals’ patients were assigned into the training and validation dataset, and the data of the remaining hospitals were assigned into the external validation dataset. The Student’s t-test and Pearson’s chi-square test were used to compare the characteristics of the training and test datasets.

The RFS and CSS rates of the training and test datasets were compared using Kaplan–Meier curves and the log-rank test. The prognostic significances of variables were calculated with the CPH model using estimated hazard ratios (HRs) with 95% confidence interval. Analyzed variables included patient age, sex, body mass index (BMI), diabetes, hypertension, Eastern Cooperative Oncology Group performance status (ECOG PS), symptoms at presentation, tumor size, pathological T stage, Fuhrman’s grade, sarcomatoid differentiation, and tumor necrosis.

Performances of the prediction models, including CPH and each machine learning model, were compared using the fivefold cross-validated Harrel’s C-index. The fivefold cross-validation technique was used in all CPH, RSF and DeepSurv training processes. In other words, out of 5 randomly divided folds, 4 folds were used as data for training, and onefold was used as data for model validation. By repeating this process 5 times, the performance of the model with the least validation loss was selected as the best optimal model. The final model’s performance was the result of the model performance which was applied to the unseen external validation dataset.

The C-index is commonly used as a metric for survival prediction and reflects how well a model predicts the ordering of event times^17^. The C-index is a generalization of the area under the receiver operating characteristic curve to regression problems and can handle right-censored data^18^. All tests were two-sided, and *p* < 0.05 was considered statistically significant. Analyses were performed using the R programming language (R Core Team, Vienna, Austria, 2018).

### Machine learning algorithm

Random forest is one of the well-known machine ensemble learning methods for classification or regression. Random forest constructs multiple tree-based classification structure and fits some decision tree classifiers using bagging and average, which can reduce the overfitting of the training data^19^. Random survival forest (RSF) is an extension of random forest for right-censored survival data^20^. RSF additionally calculates the significance of each predictor when the training dataset fits into the model, in which a high significance for some predictors means that those predictors are located in the upper root level in the tree-based structure. We used ‘randomForestSRC’ R package, and parameter settings. RSF used only important features to validate the training model. Therefore, the important variables were selected with the following parameters: ntree (number of trees to grow) = 15, mtry (number of variable randomly sampled at each split) = 12, nsplit (maximum number of split points) = 10.

DeepSurv is a package that implements a DL generalization of the CPH model using the TensorFlow structure^21^. DeepSurv uses a multilayer perceptron to self-learn the effects of a covariate. Priori selection and interaction of the covariates should be considered in designing the CPH model, but DeepSurv has the advantage of not considering this. DeepSurv is composed of 1 input layer (12 nodes for independent variables), 3 hidden layers with 6, 3, and 1 nodes with tanh activation, and output. We used the Adam optimizer with a learning rate of 0.4 and a learning rate decay of 1.0. We used dropout, batch normalization, and L1 and L2 regularization during training. We additionally experimented whether the elimination of the covariate with the least important feature leads to an improvement in the C-index. All covariates were standardized when entered into the DeepSurv model, and grid search mechanisms were used for hyperparameter optimization.

### Compliance with ethical requirements

All methods were performed in accordance with the ethical standards of the responsible committee on human experimentation (Hallym University Chuncheon Sacred Heart Hospital, and International Committee of Medical Journal Editors) and the Helsinki Declaration of 1975, as revised in 2008. Informed consent was obtained from all patients included.

## Results

### Baseline characteristics

**Figure 1:**
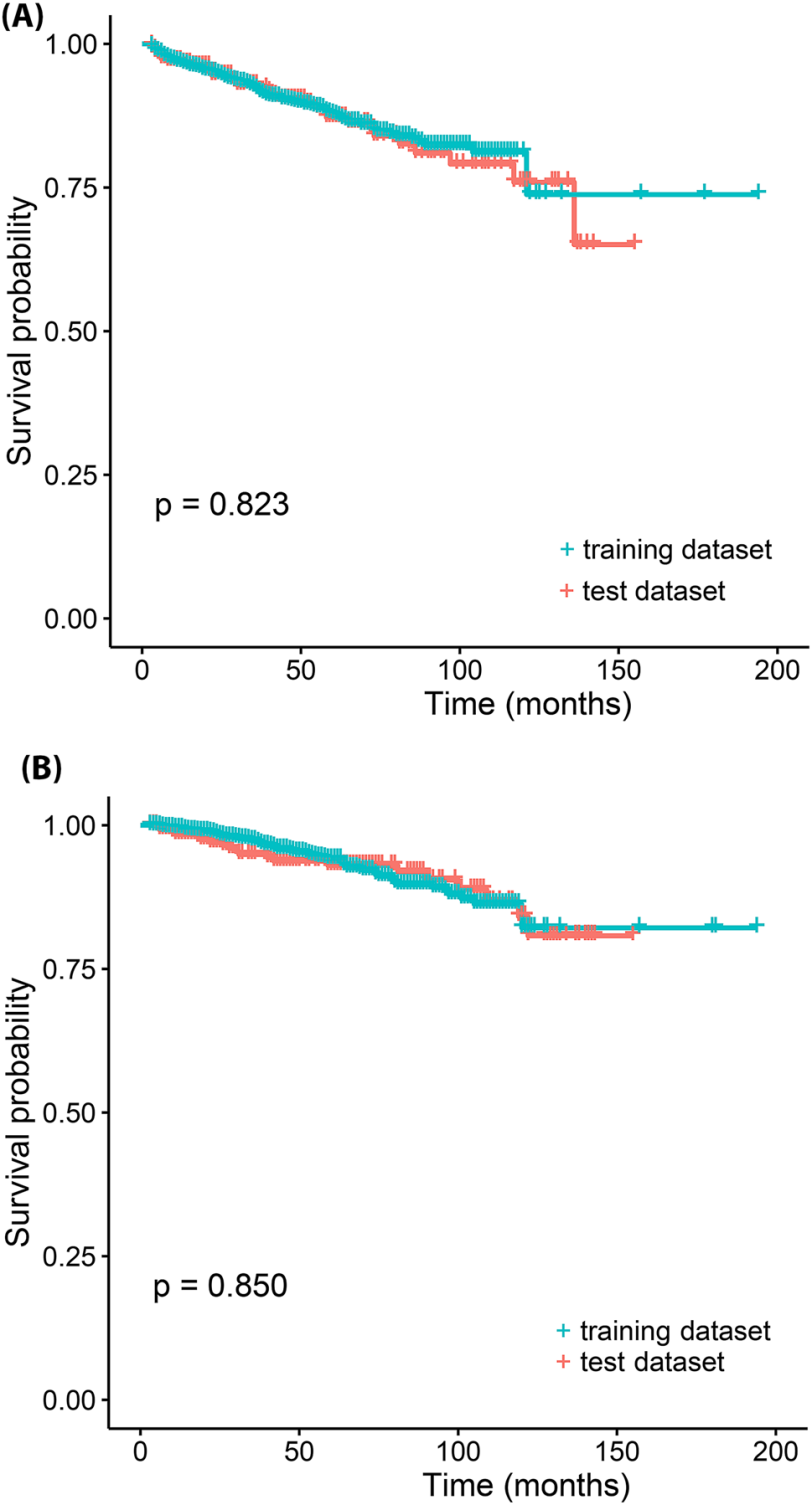
Kaplan–Meier distribution of training and test dataset of recurrence-free survival (**A**) and cancer-specific survival (**B**). Dotted line represents 95% confidence interval.

A total of 2139 nm-cRCC patients were included. Their median age was 56 years, and 1547 subjects (72.3%) were men. Table 1 shows a comparison of the clinical and pathological characteristics of the training and test datasets. Patients with male gender, high Fuhrman’s grade, or low ECOG PS were higher, and those with initial symptom were lower in the training dataset than those in the test dataset. Figure 1A shows the Kaplan–Meier RFS distribution of the training and test datasets, which were similar to each other (*p* = 0.823). The CSS distribution was also similar between the training and test datasets (*p* = 0.850) (Fig. 1B).

### Cox proportional hazard model

**Figure 2:**
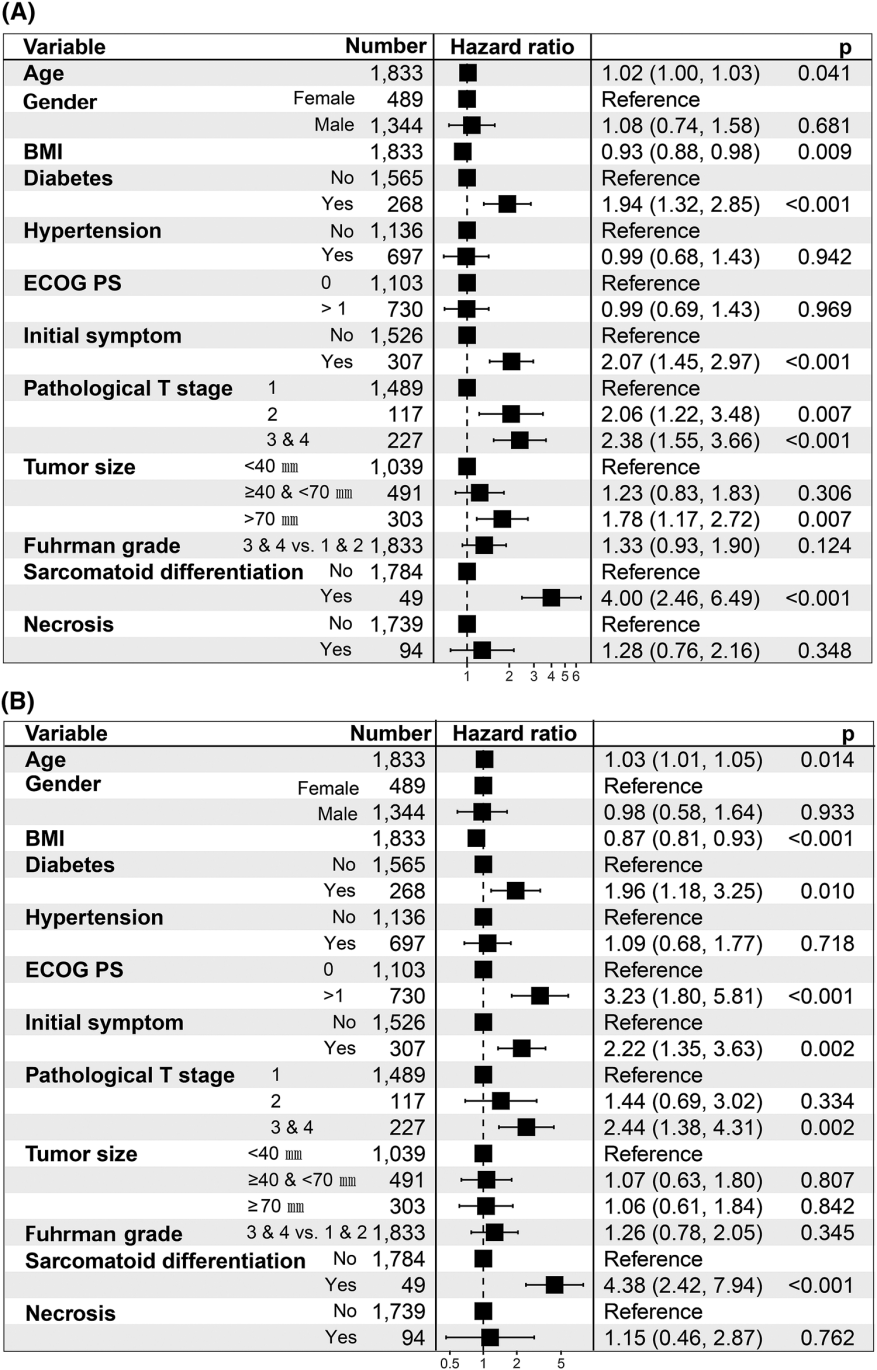
Forest plot showed the predictors of recurrence-free survival (**A**) and cancer-specific survival (**B**) of multivariable cox proportional hazard model. BMI, body mass index; ECOG PS, Eastern Cooperative Oncology Group performance status.

During the maximum of 194 months observation for the training dataset (1833 subjects), a total of 190 patients (8.9%) experienced recurrence. Those with recurrence were associated with high age (HR, 1.02; 95% CI, 1.00–1.03), low BMI (HR, 0.93; 95% CI, 0.88–0.98) and were likely to have diabetes (HR 1.94, 95% CI, 1.32–2.85), symptoms at presentation (HR, 2.07; 95% CI, 1.45–2.97) and sarcomatoid differentiation (HR 4.00, 95% CI, 2.46–6.49). Pathological T stage and tumor size also associated with recurrence in the training model (Fig. 2A). Detailed results of the univariate and multivariate CPH prediction results for RFS in the training dataset are presented in Supplemental Table 1.

One hundred and eight patients (7.0%) died of cancer-specific causes. In the multivariate CPH model of the training dataset, high BMI decreased the risk of cancer-specific death (HR, 0.87 per increase of 1 kg/m^2^; 95% CI, 0.81–0.93). Age, diabetes, high ECOG PS, symptoms at presentation, pathological T stage, and sarcomatoid differentiation were associated with increased cancer-specific death in the training dataset (Fig. 2B). Detailed results of the univariate and multivariate CPH prediction results for CSS in the training dataset are presented in Supplemental Table 2.

### Comparison of survival model performance

**Figure 3:**
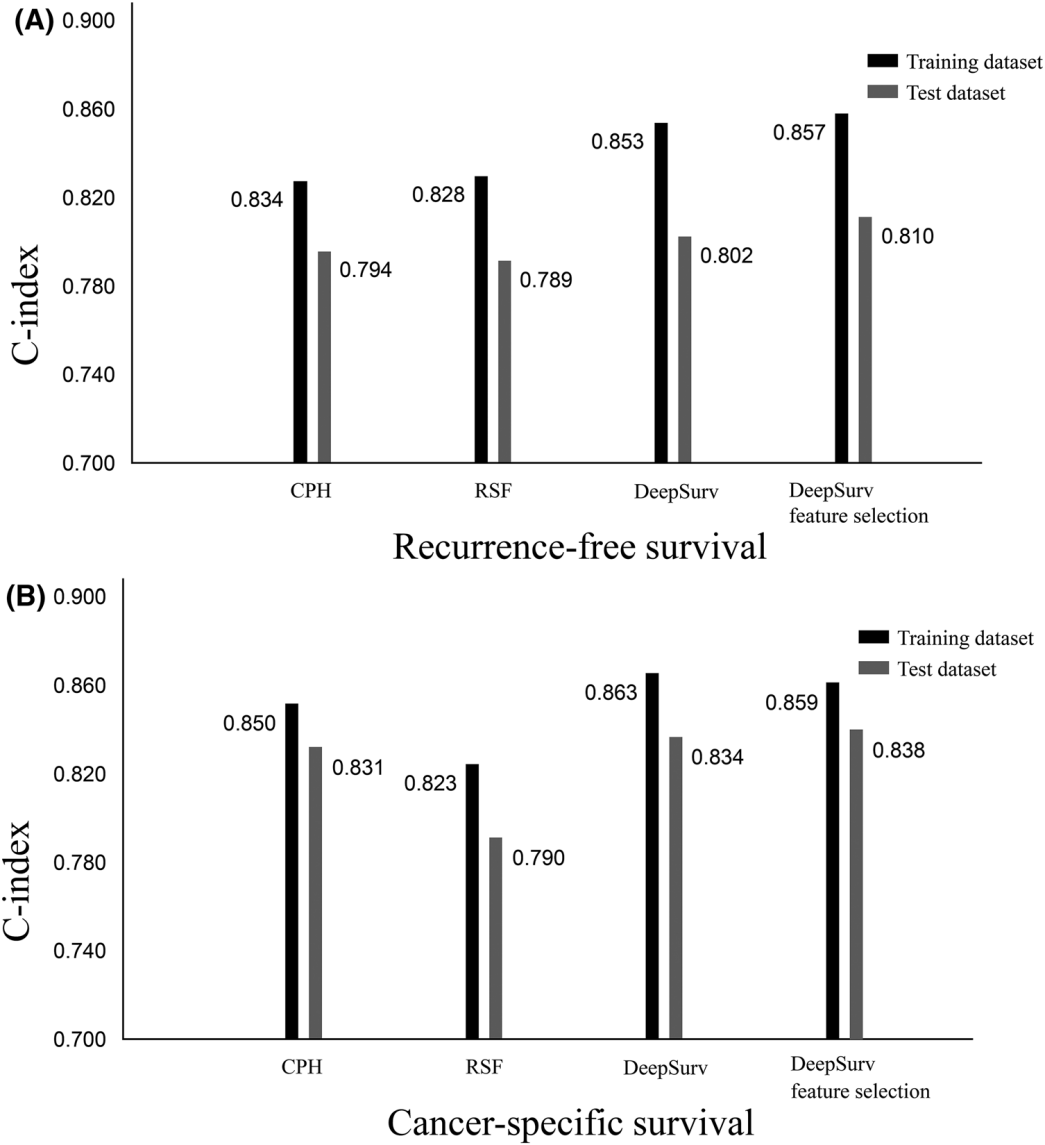
Comparison of C-indices of 3 different algorithms of recurrence-free survival (**A**) and cancer-specific survival (**B**). CPH, cox proportional hazard; RSF, random survival forest.

Figure 3 shows a comparison of C-indices between the training and test dataset using CPH, RSF, and DeepSurv algorithms. The performance of RSF for RFS (C-index = 0.789) and CSS (C-index = 0.790) was comparable to that of the CPH model (C-index = 0.794 and 0.831, respectively). The performance of DeepSurv for RFS and CSS (C-index = 0.802 and 0.834, respectively) was superior to those of CPH and RSF models. In this figure, each performance of CPH, RSF, and DeepSurv model did better on the training dataset than on the test dataset. Therefore, we additionally performed the same ML procedures for RFS with the one feature selection with grid search technique, which can reduce overfitting of the algorithms. Detailed result of DeepSurv with feature selection were showed in the supplemental Table 3. When hypertension was removed (least important covariate in feature importance) in the DeepSurv model, performance of RFS and CSS were increased to the C-index of 0.810 and 0.838, respectively. Table 2 shows the comparison of the importance of variables between the training datasets of the RSF and DeepSurv models when CSS was analyzed.

**Table 2:** Comparison of the importance of variables for cancer-specific survival in the training dataset of the random survival forest and TensorFlow DeepSurv models.

Importance	Random survival forest	DeepSurv
1st	ECOG PS	ECOG PS
2nd	Sarcomatoid differentiation	Age
3rd	Initial symptom	Pathological T stage
4th	Pathological T stage	Diabetes
5th	Diabetes	Sarcomatoid differentiation
6th	Fuhrman’s grade	Necrosis
7th	BMI	Gender
8th	Tumor size	BMI
9th	Necrosis	Tumor size
10th	Age	Fuhrman’s grade

ECOG PS, Eastern Cooperative Oncology Group performance status; BMI, body mass index.

## Discussion

Many researchers are trying to more accurately predict prognosis in RCC patients. Many anatomical, histological, clinical, and molecular markers have been introduced and studied in this field, but only the CPH model has been used for statistical analyses. Our large, multicenter cohort analysis employed DL using the DeepSurv and CPH models and demonstrated that the DeepSurv model predicted prognosis in nm-cRCC patients better than the CPH model. To our knowledge, this is the first study to assess the prognosis in RCC using a DL model, and we suggest that DL survival models are new effective tools for predicting prognosis in RCC patients.

How can we determine whether the prediction performance of DL is better than that of the CPH model? First, when evaluating the CPH model, it should always be tested whether the research hypothesis satisfies the proportional hazard assumption^22^. In other words, the CPH model assumes that the death or survival of patients is linearly related to the combination of their covariates. However, current diagnosis and treatment strategies in real practice have become more diverse and complicated than they used to be; not all covariates can always satisfy the proportional hazard assumption. When Katzman designed the DeepSurv model, the Faraggi–Simon network was reflected in the DeepSurv feed-forward neural network structure, and that is one of the non-linear extensions of the CPH model^21,23^. Therefore, DeepSurv excels in the linear and non-linear combinations of the covariates, leading to superior performance than the CPH model. Gensheimer and Narasimhan reported the performance of the Nnet, Cox-nnet, DeepSurv, and standard CPH models for predicting 1-year survival in 9105 subjects from the SUPPORT cohort^24^. They showed that cancer status (no cancer, metastatic cancer, and other cancers) violated the proportional hazards assumption in predicting survival, and the performances of DL algorithms were superior to those of the CPH model. In this regard, because DeepSurv is more optimized for non-linear algorithms than the CPH model, it can be considered to have a better performance in survival prediction. Second, DL-based algorithms such as DeepSurv are useful when dealing with large datasets compared to the CPH model. Several researchers have reported that DL-based predictions have shown excellent performances in predicting survival in hepatocellular carcinomas^13^, kidney grafts^15^, and brain glioblastomas^14^. In these reports, survival prediction performance was improved when unstructured data such as gene data, histology, or CT images were added to the model. Although unstructured data such as CT images and genetic or histologic information were not added in our analysis, we showed that covariates such as BMI or diabetes are important determinants for RSF or DeepSurv survival prediction, in addition to well-known factors related to the prognosis of cRCC. As described above, DL-based survival prediction has the advantage of discovering novel biomarkers and generating new hypotheses using large amounts of data. Of course, clinical validation is needed for these novel risk factors for DL algorithms.

In the research field of urological cancer, DL-based algorithms had been used to predict tumor grade or phenotype^25^ and to differentiate the degree of invasiveness or malignancy using genomic, histologic, and radiomics data^26,27^. Holdbrook et al.^28^ successfully quantified nuclear pleomorphic patterns using DL using cRCC pathologic slides. Other researchers have also shown that a specific texture pattern, which can be learned using DL of abdominal CT images, can successfully predict tumor grade of cRCC patients^25,29^. Ning et al. reported that image texture differentiation using convolutional neural network could be useful to classify risk of recurrence of cRCC patients^16^. This paper is similar to our works in that the prognosis of cRCC patients was predicted using DL. However, we performed DL task using numerical data rather than image data. In addition, there was no important information for the judgement of predicting high risk population in this previous work, we could provide information on what variable were important to predict the outcomes. From the viewpoint of precision medicine, an individual's disease-related risks should be estimated using variety of data, including genetic analysis, advanced imaging, and individual health-related lifelogs. To construct a model that reflects such massive data and data that can change the risks over time, DL-based survival models can be applied and gradually improved to deal with vast and non-linear real-world situations. In this regard, it is noted that ours is the first study to assess RCC prognosis using a DL model.

It is interesting that ECOG PS and age was the most important variable for CSS in TensorFlow DeepSurv model, contrary to general expectations. Unlike the CPH model which only considers linear combinations of variables, DeepSurv model considers non-linear combinations as well as linear combinations^21,23^. This result may be due to structural differences between the two models.

Our study has a few limitations. First, the study design was retrospective in nature. Second, potential prognostic factors such as molecular markers were not assessed. These factors might lead to better prediction performance for RCC prognosis. However, the most widely used conventional prognostic factors of nm-cRCC were assessed. Third, our study lacked a central pathological review, which may result in misclassifications or misdiagnoses due to inter-observer variability. However, pathological assessments were performed by urological pathologists at each institution.

## Conclusions

We presented a promising DL prediction algorithm for survival of nm-cRCC. For predicting RFS and CSS in nm-cRCC patients, DeepSurv showed superior performance than CPH or RSF. Soon, DL-based survival prediction for RCC patients will be useful when dealing with large amounts of histological or imaging data or when considering the effects of covariates over time.

## Supplementary Information

Supplementary Table 1.

Supplementary Table 2.

Supplementary Table 3.
