# Supplementary material for: Deep learning based prediction of prognosis in nonmetastatic clear cell renal cell carcinoma
Source: Sci Rep. 2021 Jan 13;11:1242. doi: 10.1038/s41598-020-80262-9 (PMC7806580; doi:10.1038/s41598-020-80262-9)
Supplement: Supplementary file 1 — Supplementary Table 1. [file 41598_2020_80262_MOESM1_ESM.docx]

**Deep learning based prediction of prognosis in nonmetastatic clear cell renal cell carcinoma**

**Running title:** Deep learning survival in nm-cRCC

Seok-Soo Byun^1^, Tak Sung Heo^2^, Jeong Myeong Choi^2^, Yeong Seok Jeong^3^, Yu Seop Kim^3^, Won Ki Lee^4^* & Chulho Kim^5,6^*

^1^Department of Urology, Seoul National University Bundang Hospital, Seongnam, Korea

^2^Department of Convergence Software, Hallym University, Chuncheon, Korea

^3^College of Software, Hallym University, Chuncheon, Korea

^4^Department of Urology, Hallym University Chuncheon Sacred Heart Hospital, Chuncheon, Korea

^5^Department of Neurology, Hallym University Chuncheon Sacred Heart Hospital, Chuncheon, Korea

^6^Chuncheon Translational Research Center, Hallym University, Chuncheon, Korea

***Corresponding Authors (co-corresponding) :**

Won Ki Lee,

Department of Urology, College of Medicine, Hallym University, Chuncheon Sacred Hospital, 153, Kyo-dong, Chuncheon, Korea

Tel: 82-33-240-5161; Fax: 82-33-240-5426; E-mail: rheewk@hanmail.net

Chulho Kim,

Department of Neurology, College of Medicine, Hallym University, Chuncheon Sacred Hospital, 153, Kyo-dong, Chuncheon, Korea

Tel: 82-33-240-5255; Fax: 82-33-255-6244; E-mail: gumdol52@hallym.or.kr

|  | Univariate | |  | Multivariate | |
| --- | --- | --- | --- | --- | --- |
|  | HR (95% CI) | P value |  | HR (95% CI) | P value |
| Age | 1.03 (1.02–1.04) | <0.001 |  | 1.02 (1.00–1.03) | 0.041 |
| Gender : Male vs. Female | 1.09 (0.76–1.56) | 0.659 |  | 1.08 (0.74–1.58) | 0.681 |
| BMI | 0.93 (0.88–0.97) | 0.002 |  | 0.93 (0.88–0.98) | 0.009 |
| Diabetes | 2.41 (1.69–3.44) | <0.001 |  | 1.94 (1.32–2.85) | <0.001 |
| Hypertension | 1.51 (1.10–2.08) | 0.010 |  | 0.99 (0.68–1.43) | 0.942 |
| ECOG PS : ≥ 1 vs. 0 | 1.33 (0.97–1.82) | 0.073 |  | 0.99 (0.69–1.43) | 0.969 |
| Symptoms at presentation | 3.89 (2.84–5.33) | <0.001 |  | 2.07 (1.45–2.97) | <0.001 |
| T stage 1 | 1.00 (–) | reference |  | 1.00 (–) | Reference |
| 2 | 3.88 (2.48–6.07) | <0.001 |  | 2.06 (1.22–3.48) | 0.007 |
| 3 and 4 | 5.59 (3.94–7.93) | <0.001 |  | 2.38 (1.55–3.66) | <0.001 |
| Tumor size < 40mm | 1.00 (–) | reference |  | 1.00 (–) | Reference |
| ≥ 40 and < 70mm | 1.76 (1.20–2.58) | 0.004 |  | 1.23 (0.83–1.83) | 0.306 |
| ≥ 70 mm | 3.23 (2.22–4.70) | <0.001 |  | 1.78 (1.17–2.72) | 0.007 |
| Fuhrman’s grade : 3 and 4 vs. 1 and 2 | 2.45 (1.78–3.38) | <0.001 |  | 1.33 (0.93–1.90) | 0.124 |
| Sarcomatoid differentiation | 12.24 (8.06–18.60) | <0.001 |  | 4.00 (2.46–6.49) | <0.001 |
| Tumor necrosis | 4.18 (2.78–6.29) | <0.001 |  | 1.28 (0.76–2.16) | 0.348 |

**Supplemental Table 1.** Determinant of recur-free survival of Cox proportional hazard model in the training dataset.

BMI, body mass index; HR, hazard ratio; CI, confidence interval.
