# Supplementary material for: Deep learning based prediction of prognosis in nonmetastatic clear cell renal cell carcinoma
Source: Sci Rep. 2021 Jan 13;11:1242. doi: 10.1038/s41598-020-80262-9 (PMC7806580; doi:10.1038/s41598-020-80262-9)
Supplement: Supplementary file 2 — Supplementary Table 2. [file 41598_2020_80262_MOESM2_ESM.docx]

**Deep learning based prediction of prognosis in nonmetastatic clear cell renal cell carcinoma**

**Running title:** Deep learning survival in nm-cRCC

Seok-Soo Byun^1^, Tak Sung Heo^2^, Jeong Myeong Choi^2^, Yeong Seok Jeong^3^, Yu Seop Kim^3^, Won Ki Lee^4^* & Chulho Kim^5,6^*

^1^Department of Urology, Seoul National University Bundang Hospital, Seongnam, Korea

^2^Department of Convergence Software, Hallym University, Chuncheon, Korea

^3^College of Software, Hallym University, Chuncheon, Korea

^4^Department of Urology, Hallym University Chuncheon Sacred Heart Hospital, Chuncheon, Korea

^5^Department of Neurology, Hallym University Chuncheon Sacred Heart Hospital, Chuncheon, Korea

^6^Chuncheon Translational Research Center, Hallym University, Chuncheon, Korea

***Corresponding Authors (co-corresponding) :**

Won Ki Lee,

Department of Urology, College of Medicine, Hallym University, Chuncheon Sacred Hospital, 153, Kyo-dong, Chuncheon, Korea

Tel: 82-33-240-5161; Fax: 82-33-240-5426; E-mail: rheewk@hanmail.net

Chulho Kim,

Department of Neurology, College of Medicine, Hallym University, Chuncheon Sacred Hospital, 153, Kyo-dong, Chuncheon, Korea

Tel: 82-33-240-5255; Fax: 82-33-255-6244; E-mail: gumdol52@hallym.or.kr

**Supplemental Table 2.** Determinant of cancer-specific survival of Cox proportional hazard model in the training dataset.

|  | Univariate | |  | Multivariate | |
| --- | --- | --- | --- | --- | --- |
|  | HR (95% CI) | P value |  | HR (95% CI) | P value |
| Age | 1.04 (1.02–1.06) | <0.001 |  | 1.03 (1.01–1.05) | 0.014 |
| Gender : Male vs. Female | 1.05 (0.65–1.70) | 0.853 |  | 0.98 (0.58–1.64) | 0.933 |
| BMI | 0.88 (0.82–0.94) | <0.001 |  | 0.87 (0.81–0.93) | <0.001 |
| Diabetes | 3.33 (2.13–5.21) | <0.001 |  | 1.96 (1.18–3.25) | 0.010 |
| Hypertension | 1.88 (1.24–2.86) | 0.001 |  | 1.09 (0.68–1.77) | 0.718 |
| ECOG PS : ≥ 1 vs. 0 | 4.61 (2.80–7.59) | <0.001 |  | 3.23 (1.80–5.81) | <0.001 |
| Symptoms at presentation | 4.60 (3.02–6.98) | <0.001 |  | 2.22 (1.35–3.63) | 0.002 |
| T stage 1 | 1.00 (–) | reference |  | 1.00 (–) | reference |
| 2 | 2.81 (1.45–5.48) | 0.002 |  | 1.44 (0.69–3.02) | 0.334 |
| 3 and 4 | 6.99 (4.47–10.95) | <0.001 |  | 2.44 (1.38–4.31) | 0.002 |
| Tumor size < 40mm | 1.00 (–) | reference |  | 1.00 (–) | reference |
| ≥ 40 and < 70mm | 1.47 (0.89–2.42) | 0.130 |  | 1.07 (0.63–1.80) | 0.807 |
| ≥ 70 mm | 2.09 (1.25–3.48) | 0.005 |  | 1.06 (0.61–1.84) | 0.842 |
| Fuhrman’s grade : 3 and 4 vs. 1 and 2 | 2.44 (1.59–3.75) | <0.001 |  | 1.26 (0.78–2.05) | 0.345 |
| Sarcomatoid differentiation | 16.77 (10.38–27.02) | <0.001 |  | 4.38 (2.42–7.94) | <0.001 |
| Tumor necrosis | 1.40 (0.65–3.03) | 0.395 |  | 1.15 (0.46–2.87) | 0.762 |

BMI, body mass index; HR, hazard ratio; CI, confidence interval.
