# Supplementary material for: Deep learning based prediction of prognosis in nonmetastatic clear cell renal cell carcinoma
Source: Sci Rep. 2021 Jan 13;11:1242. doi: 10.1038/s41598-020-80262-9 (PMC7806580; doi:10.1038/s41598-020-80262-9)
Supplement: Supplementary file 3 — Supplementary Table 3. [file 41598_2020_80262_MOESM3_ESM.docx]

**Deep learning based prediction of prognosis in nonmetastatic clear cell renal cell carcinoma**

**Running title:** Deep learning survival in nm-cRCC

Seok-Soo Byun^1^, Tak Sung Heo^2^, Jeong Myeong Choi^2^, Yeong Seok Jeong^3^, Yu Seop Kim^3^, Won Ki Lee^4^* & Chulho Kim^5,6^*

^1^Department of Urology, Seoul National University Bundang Hospital, Seongnam, Korea

^2^Department of Convergence Software, Hallym University, Chuncheon, Korea

^3^College of Software, Hallym University, Chuncheon, Korea

^4^Department of Urology, Hallym University Chuncheon Sacred Heart Hospital, Chuncheon, Korea

^5^Department of Neurology, Hallym University Chuncheon Sacred Heart Hospital, Chuncheon, Korea

^6^Chuncheon Translational Research Center, Hallym University, Chuncheon, Korea

***Corresponding Authors (co-corresponding) :**

Won Ki Lee,

Department of Urology, College of Medicine, Hallym University, Chuncheon Sacred Hospital, 153, Kyo-dong, Chuncheon, Korea

Tel: 82-33-240-5161; Fax: 82-33-240-5426; E-mail: rheewk@hanmail.net

Chulho Kim,

Department of Neurology, College of Medicine, Hallym University, Chuncheon Sacred Hospital, 153, Kyo-dong, Chuncheon, Korea

Tel: 82-33-240-5255; Fax: 82-33-255-6244; E-mail: gumdol52@hallym.or.kr

**Supplemental Table 3.** Result of C-index of DeepSurv for recurrence free survival with feature selection.

| Except covariates | Training C-index | Test C-Index | Loss | Covariates importance Rank |
| --- | --- | --- | --- | --- |
| Nothing | 0.858855 | 0.779663 | 5.6034 | X |
| Age | 0.848121 | 0.757254 | 5.6347 | 2 |
| BMI | 0.864046 | 0.772823 | 5.6905 | 8 |
| Diabetes | 0.859640 | 0.766962 | 5.6820 | 4 |
| Hypertension | 0.857324 | 0.809737 | 5.1863 | 12 |
| Initial symptom (Yes vs. No) | 0.847993 | 0.789689 | 5.6993 | 11 |
| T stage (1 vs. 2 vs. 3,4) | 0.865525 | 0.758571 | 5.4979 | 3 |
| Tumor grade (3,4 vs. 1,2) | 0.851893 | 0.781114 | 5.6330 | 10 |
| Sarcomatoid differentiation | 0.857553 | 0.767521 | 5.6571 | 5 |
| Necrosis | 0.860129 | 0.770523 | 5.6124 | 6 |
| Tumor size (< 40mm vs. ≥ 40 and < 70mm vs. ≥ 70 mm) | 0.852488 | 0.775211 | 5.4804 | 9 |
| Gender (Female vs. Male) | 0.858411 | 0.771689 | 5.4513 | 7 |
| ECOG PS (≥ 1 vs. 0) | 0.773332 | 0.751304 | 5.9969 | 1 |

BMI, body mass index; ECOG PS, Eastern Cooperative Oncology Group performance status.
